# NBEAL1 controls SREBP2 processing and cholesterol metabolism and is a susceptibility locus for coronary artery disease

**DOI:** 10.1038/s41598-020-61352-0

**Published:** 2020-03-11

**Authors:** Christian Bindesbøll, Aleksander Aas, Margret Helga Ogmundsdottir, Serhiy Pankiv, Trine Reine, Roberto Zoncu, Anne Simonsen

**Affiliations:** 1 Department of Molecular Medicine, Institute of Basic Medical Sciences and Centre for Cancer Cell Reprogramming, Institute of Clinical Medicine, Faculty of Medicine, University of Oslo, 1112 Blindern, 0317 Oslo, Norway; 2 Department of Biochemistry and Molecular Biology, Biomedical Center, Faculty of Medicine, University of Iceland, Vatnsmyrarvegur 16, 101 Reykjavik, Iceland; 3 Department of Nutrition, Institute of Basic Medical Sciences, University of Oslo, 1112 Blindern, 0317 Oslo, Norway; 4 Section for Interphase genetics, Institute for Cancer Genetics and Informatics, Oslo University Hospital, 0424 Oslo, Norway; 5 Department of Molecular and Cell Biology, University of California, Berkeley, Berkeley, CA 94720 USA

**Keywords:** Mechanisms of disease, Gene expression

## Abstract

Dysregulated cholesterol homeostasis promotes the pathology of atherosclerosis, myocardial infarction and strokes. Cellular cholesterol is mainly regulated at the transcriptional level by SREBP2, but also through uptake of extracellular cholesterol from low density lipoproteins (LDL) via expression of LDL receptors (LDLR) at the cell surface. Identification of the mechanisms involved in regulation of these processes are thus key to understand the pathology of coronary artery disease. Here, we identify the large and poorly characterized BEACH domain protein Neurobeachin-like (NBEAL) 1 as a Golgi- associated protein required for regulation of cholesterol metabolism. *NBEAL1* is most abundantly expressed in arteries. Genetic variants in *NBEAL1* are associated with decreased expression of *NBEAL1* in arteries and increased risk of coronary artery disease in humans. We show that NBEAL1 regulates cholesterol metabolism by modulating LDLR expression in a mechanism involving interaction with SCAP and PAQR3 and subsequent SREBP2-processing. Thus, low expression of *NBEAL1* may lead to increased risk of coronary artery disease by downregulation of LDLR levels.

## Introduction

NBEAL1 belongs to a family of proteins that shares a highly conserved domain known as the BEACH (Beige and Chediak-Higashi) domain, found in nine human proteins. The cellular functions of most BEACH proteins still remain poorly defined more than a decade after the crystal structure of the BEACH domain was resolved^1,2^, but several family members are linked to membrane trafficking and/or modeling processes, including regulation of lysosome size, autophagy, apoptosis and granule size^3–6^. Variants in genes encoding distinct BEACH proteins cause several human diseases, including grey platelet syndrome (NBEAL2)^7–9^, Chédiak-Higashi Syndrome (LYST)^10^ and human primary microcephaly (ALFY/WDFY3)^11^. NBEAL1 is one of the least understood BEACH proteins and we therefore aimed to elucidate its cellular localization and function, as well as a potential link to disease.

## Results and Discussion

**Figure 1:**
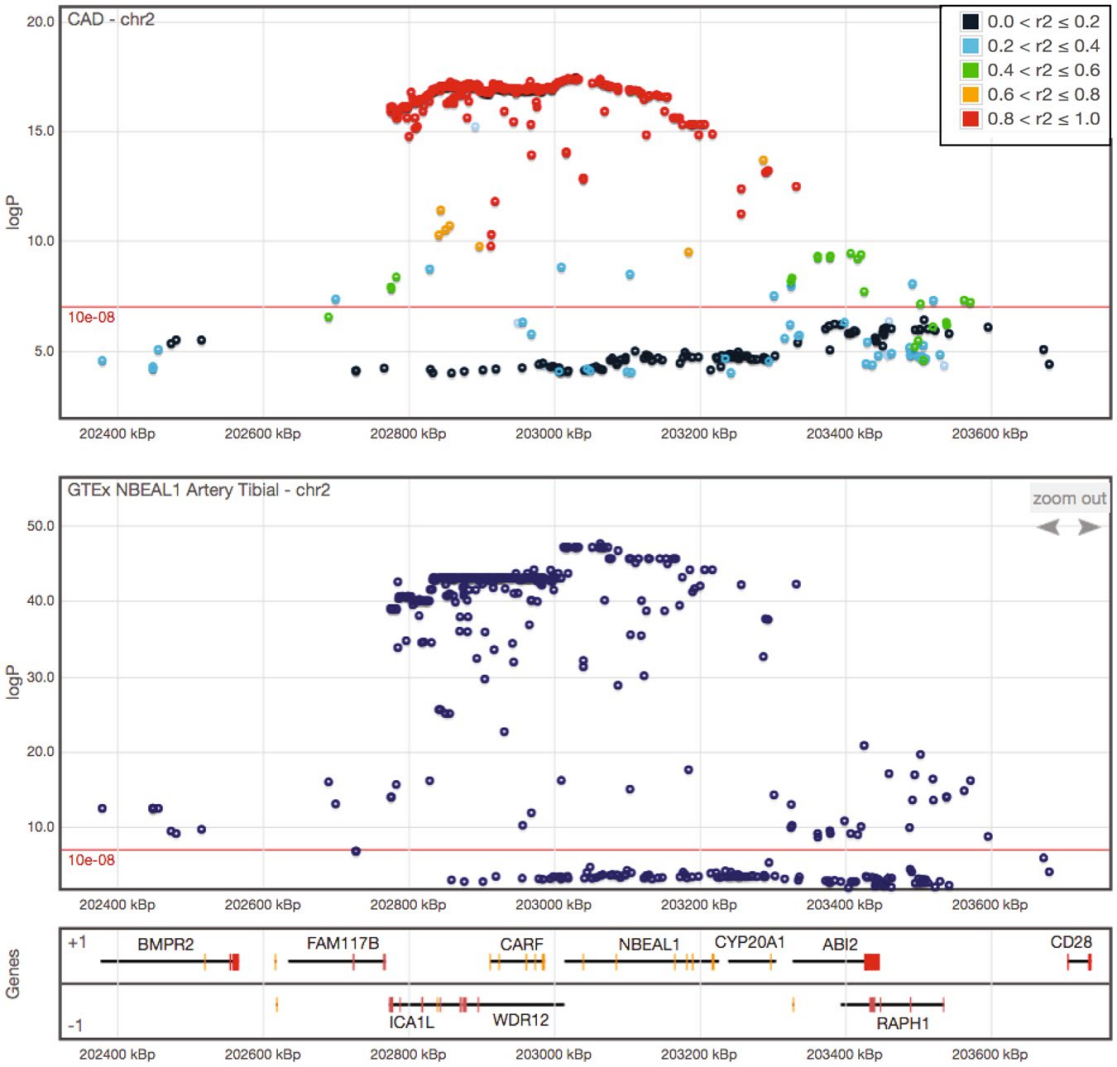
Genetic variants in *NBEAL1* are associated with increased risk of coronary artery disease and decreased *NBEAL1* expression. Regional association plots of *NBEAL1* and nearby genes. The top plot shows associations with CAD (CARDIoGRAMplusC4D data) and the bottom plot shows expression of *NBEAL1* in Artery Tibial (GTEx data). SNPs are plotted with their analysis P values (as −log10 values) against their genomic position (NCBI Build 38). The local LD structure relative to the top CAD association is displayed on the top plot, with the different colors representing the strength of the correlation (based on pairwise r2 values from 1000 G EUR).

The *NBEAL1* locus has previously been associated with coronary artery disease (CAD) (also called coronary heart disease or coronary atherosclerosis)^12,13^. However, the culprit at the locus in relation to CAD has remained somewhat unclear. In order to characterize a possible role of NBEAL1 in the disease, we utilized data from the CAD Genome-wide Replication and Meta-analysis (CARDIoGRAMplusC4D) consortium that holds information on genome wide association data for CAD comprising 60,801 cases and 123,504 controls^14,15^. *NBEAL1* is located on chromosome 2 and using this data we performed a lookup of variants on this chromosome that associated with CAD (Supplemental Table 1). Interestingly, this revealed the strongest association within chromosome 2 of an intron variant in *NBEAL1* with 1.15-fold increased risk of CAD in carriers, present in 13% of Europeans (rs115654617, OR 1.15, MAF 13%; *p* = 3.12 × 10^−18^). This variant is among 291 variants that all associate with CAD (p < 1 × 10^−8^) within a 1.2 Mb distance from *NBEAL1* (Fig. 1, Supplemental Table 1). We assessed whether these variants are inherited together more often than random by estimating their linkage disequilibrium (LD) and found that of these 291 variants, 207 are in LD with the *NBEAL1* variant rs115654617 (r2 > 0.8; Fig. 1, Supplemental Table 2). Among the linked variants is a previously reported variant associated with increased risk of early onset myocardial infarction, (*WDR12*, rs6725887)^12^, and a previously reported variant associated with white matter hyperintensity volumes in stroke patients (*NBEAL1*, rs72934505)^13^. Moreover, a recent study connected a variant in *NBEAL1* with increased atherosclerotic lesions in young persons, but this did not reach genome-wide significance^16^. Gene expression of *NBEAL1* has also been reported to increase in the brain of patients with glioma compared to healthy controls^17^, and NBEAL1 was recently identified as a candidate risk gene for hereditary breast cancer^18^.

The prevalence and importance of disease-related genetic variants in non-protein-coding regions of the genome is well documented^19,20^. These variants may regulate how and when a gene is expressed^20^. Interestingly, analysis of RNA sequencing data from different human tissues held by the GTEx Portal database revealed highest expression of *NBEAL1* in arteries (Supplemental Fig. 1a)^21^. According to the Ensembl database and GTEx RNA sequencing, three protein coding isoforms of *NBEAL1* exist, where the longest isoform (ENST00000449802) showed highest expression in all tissues, including arteries (Supplemental Fig. 1b,c). Utilizing the GTEx Portal database, we analyzed a possible association of *NBEAL1* rs115654617 with altered gene expression in various tissues. Interestingly, the variant associates strongly with decreased expression of *NBEAL1* in arteries, but also in adipose tissue, nerve, lung and skin (Supplemental Fig. 2). Moreover, the *NBEAL1* variant also associates with altered expression of nearby genes (*ICA1L*, *CARF*, *FAM117B*) in various tissues (Supplemental Table 3). Analysing the nearby 291 variants associated with CAD, expression data was available in the GTEx Portal database for 281 of the variants. Interestingly, these linked variants both associate with increased CAD risk and decreased *NBEAL1* expression in arteries (Fig. 1, Supplemental Table 4). Taken together, we find that linked CAD variants in *NBEAL1* and nearby genes associate with decreased expression of *NBEAL1* in arteries. The causal variant remains to be elucidated and whether the genetic associations are explained by the same causal variant. However, the analysis suggests that low *NBEAL1* expression in arteries confers an increased risk of CAD.

**Figure 2:**
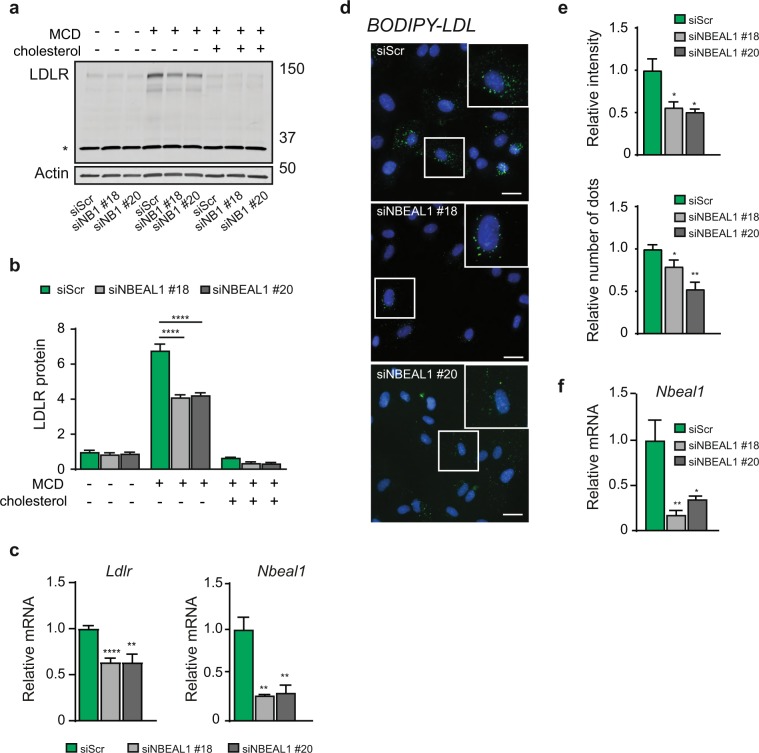
NBEAL1 modulates expression of LDL receptor and LDL uptake. (**a**) HUVEC cells depleted of NBEAL1 using two different siRNAs were sterol-depleted for 2 h using 0.5% MCD or depleted and, where indicated, restimulated with cholesterol for 6 h followed by western blotting with indicated antibodies. Asterisk denotes unspecific band (N = 3). (**b**) Quantification of LDLR relative to Actin in HUVEC cells treated as in (**a**) from three independent experiments. All bars show mean ± S.E.M. ****P < 0.0001 by two-way ANOVA followed by Tukey’s multiple comparison test. (**c**) Gene expression of NBEAL1 from (**a**) presented as mean ± S.E.M. Statistical differences were analyzed using one-way ANOVA followed by Dunnett’s multiple comparison test relative to siScr. **P < 0.001, ****P < 0.0001 (N = 3). (**d**) Representative images of 10 min BODIPY-LDL uptake in HUVECs with and without depletion of NBEAL1 using two different siRNAs. Cells were grown in lipid starved medium for 18 h prior to BODIPY-LDL-uptake. Nuclei are stained with DAPI (blue). Scale bars, 40 µM (N = 3). (**e**) Quantification of BODIPY-LDL uptake in NBEAL1 depleted HUVEC cells (**e**), displayed as average intensity per cell (upper panel) and average number of dots (lower panel). Data show mean ± S.E.M. relative to control from three independent experiments. On average 500 cells from each condition was quantified per experiment using CellProfiler. *P < 0.05, **P < 0.01 by Student’s t-test relative to control. (**f**) Gene expression of NBEAL1 from (**d**) presented as mean ± S.E.M. Statistical differences were analyzed using one-way ANOVA followed by Dunnett’s multiple comparison test relative to siScr. *P < 0.05, **P < 0.01 (N = 3).

Vascular endothelial cells form an inner lining of the blood vessels and regulate exchanges between the bloodstream and the surrounding tissues. Endothelial dysfunction is implicated in atherosclerosis and several intervention studies with effect on cardiovascular risk factors also regulate endothelial function and cardiovascular outcomes^22^. To better understand the link between decreased expression of *NBEAL1* in arteries and increased risk of CAD we tested if depletion of NBEAL1 in primary endothelial cells (human umbilical vein endothelial cells; HUVECs) would modulate expression of adhesion molecules or cholesterol metabolism in these cells. The expression of vascular adhesion molecule 1 (VCAM-1) and E-selectin is increased in vascular endothelial cells following inflammation, leading to monocyte recruitment^23^, a hallmark of early stages of artheriosclerosis^24,25^, but are also expressed in early artheriosclerotic lesions^26,27^ and play a key role in the pathology of artheriosclerosis and cardiovascular disease^28–30^. The expression of both VCAM-1 and E-selectin was robustly increased following treatment with the proinflammatory cytokine interleukin-1β (IL-1β), but there were no differences between control cells and cells depleted of NBEAL1 (Supplemental Fig. 3a–c). Depletion of RELA (also known as nuclear factor NF-kappa-B p65 subunit) was used as a positive control as VCAM-1 and E- selectin are well characterized NF-kappa-B target genes. Thus, NBEAL1 does not seem to be important for recruiting monocytes to the endothelial cells in the arteries during inflammatory conditions.

Elevated plasma levels of LDL cholesterol are associated with increased risk of CAD^31^. LDLR mediates cellular LDL uptake and low levels or loss-of-function mutations in LDLR leads to increased plasma LDL and subsequent premature CAD^31^. Because low expression of NBEAL1 was associated with increased risk of CAD, we tested if depletion of NBEAL1 could modulate LDLR levels. Interestingly, the expression of LDLR was lower in primary endothelial HUVECs depleted of NBEAL1 (using two different siRNA oligos) as compared to control cells starved of cholesterol with methyl-β cyclodextrin (MCD), while no significant difference was observed in untreated control cells or upon cholesterol replenishment following cholesterol starvation (Fig. 2a–c). In line with this, LDL uptake was inhibited in serum starved HUVECs depleted of NBEAL1 as compared to control cells (Fig. 2d–f).

**Figure 3:**
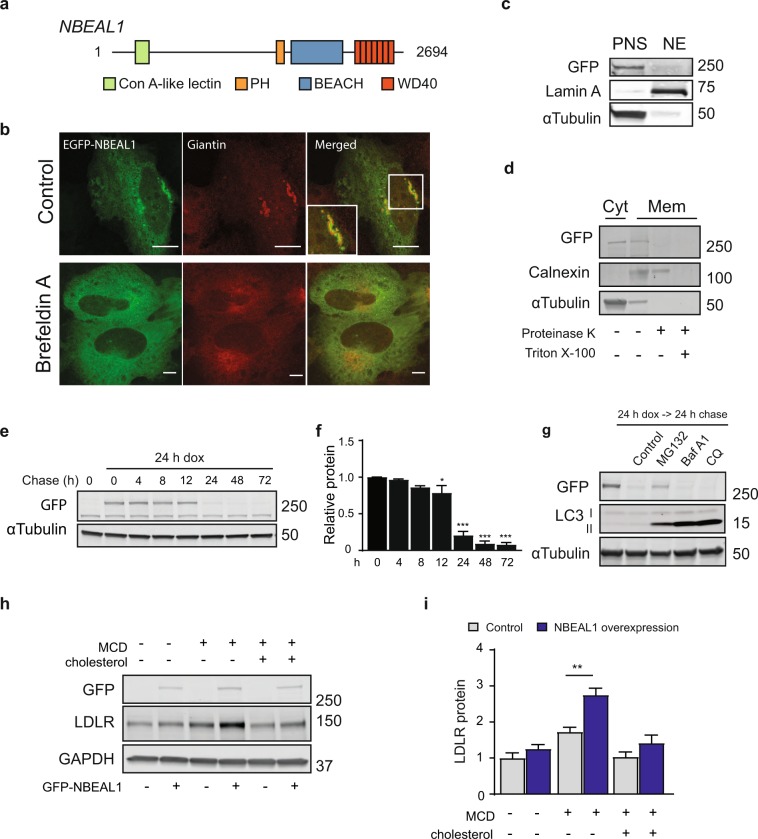
NBEAL1 is a Golgi-associated protein that is degraded by the proteasome. (**a**) Schematic representation of full-length human NBEAL1. Key domains of the protein are highlighted with boxes including the Concanavalin A (ConA)-like lectin, the pleckstrin homology (PH), BEACH, and WD40 repeat domains. The numbers refer to amino acids. Seven predicted WD40 domains following the NBEAL1 BEACH domain by using the RaptorX 3D model prediction program^33^. These formed a predicted circularized β-propeller structure. (**b**) EGFP-NBEAL1 expressing U2OS cells were treated or not with Brefeldin A for 10 min (5 µg/mL final), fixed and subjected to immunofluorescence staining of endogenous Giantin. Scale bars, 10 µm (N = 3). (**c**) Postnuclear supernatant (PNS) and nuclear (NE) fractions from EGFP-NBEAL1 expressing HEK-293T cells were immunoblotted with a GFP antibody using Lamin A or α-Tubulin as loading controls (N = 3). (**d**) Cytosolic (Cyt) or membrane (Mem) fractions from EGFP-NBEAL1 expressing U2OS cells were treated or not with proteinase K and Triton- X 100 as indicated. Membranes were immunoblotted with a GFP antibody using Calnexin or α-Tubulin as cellular fraction controls (N = 3), and Calnexin as a control that was protected upon proteinase K treatment alone, but not in combination with 1% Triton X-100. (**e**) EGFP-NBEAL1 expressing HEK-293T cells were treated with or without doxycycline (dox) for 24 h and then chased in complete media without doxycycline for the indicated time points (N = 3). (**f**) Quantified EGFP-NBEAL1 to Actin from three independent experiments. All bars show mean ±S.E.M. *P < 0.05, ***P < 0.0001 by one-way ANOVA followed by Dunnett’s multiple comparison relative to 0 h. (**g**) EGFP-NBEAL1 expressing HEK- 293T cells were treated or not with dox for 24 h, then chased in complete media in the presence or absence of 100 nM MG-132, 100 nM Bafilomycin A1 (Baf) or 50 µM chloroquine (CQ) for 24 h without dox (N = 2). Cell lysates were immunoblotted with the indicated antibodies. LC3-II is mainly degraded through autophagy and was used as a control for BafA1 and CQ treatment. (**h**) EGFP-NBEAL1 HEK-293T cells were sterol-depleted for 2 h using 0.5% MCD or depleted and, where indicated, restimulated with cholesterol for 6 h following western blotting with indicated antibodies (N = 3). (**i**) Quantification of LDLR relative to GAPDH in HEK-293T expressing EGFP-NBEAL1 cells treated as in (**c**) from three independent experiments. All bars show mean ± S.E.M. **P < 0.01 by two-way ANOVA followed by Tukey’s multiple comparison test.

No commercial antibodies exist for NBEAL1 and despite several attempts (Supplemental Fig. 4a) we failed to generate specific NBEAL1 antibodies. To identify the cellular localization and function of NBEAL1 we therefore cloned full length NBEAL1 from U2OS cell cDNA and generated cell lines (T-Rex-Flp-In doxycycline-inducible U2OS, HEK-293T and HeLa cells) with stable inducible expression of EGFP-NBEAL1. NBEAL1 has the same modular structure as most of the other BEACH proteins with a C-terminal pleckstrin homology (PH), BEACH and WD40 repeat domain assembly, and contains an additional Concanavalin A (ConA)-like lectin domain (Fig. 3a). The cloned sequence was identical to NM_001114132.1 (9058 base pairs), but lacked base pair 639–848 corresponding to exon 5–6 (amino acids 10–172) in the predicted sequence, still preserving the predicted modular structure. We identified this isoform, and several other alternatively spliced isoforms of NBEAL1 by cloning and sequencing the 5′ end of NBEAL1 form U2OS cells cDNA (Supplemental Fig. 4b). We also observed several NBEAL1 isoforms in the other cell lines used in this study (HeLa, HEK-293T, HUVECs), indicating the presence of multiple transcripts of NBEAL1. The functional significance of the different isoforms remains to be elucidated.

The modular structure of NBEAL1 does not provide many functional clues because the ConA, PH and WD40 domains are found in a wide range of proteins implicated in different cellular pathways and the function of the BEACH domain remains unknown. Of the nine BEACH proteins, NBEAL1 is most closely related to NBEAL2^6^, but there is no evidence suggesting that they have similar functions. U2OS cells are well suited for imaging and stably expressed EGFP-NBEAL1 displayed partly diffuse cytoplasmic staining in addition to localization to the Golgi compartment, as visualized by co-localization with the Golgi marker Giantin and a more diffuse staining following Brefeldin A treatment to disrupt Golgi (Fig. 3b). A similar localization was seen in cells transiently overexpressing 3xFLAG-NBEAL1 or mCherry-NBEAL1 (Supplemental Fig. 5), suggesting that EGFP-NBEAL1 is either tethered to or localized within the Golgi. EGFP-NBEAL1 was detected as a single band above 250 kDa in the post nuclear supernatant fraction (Fig. 3c) and was present both in the cytosol and membrane fraction of U2OS cells (Fig. 3d). NBEAL1 was not protected from proteinase K (Fig. 3d), indicating it is associated to and not localized within the Golgi compartment. To study the half-life of EGFP-NBEAL1 in HEK-293T cells, protein expression was induced by doxycycline and followed over 72 h after removal of doxycycline (Fig. 3e). More than 70% of EGFP-NBEAL1 was degraded after 24 h (Fig. 3e,f), which was prevented by inhibitors of proteasomal (MG-132), but not autophagic (Bafilomycin A1 (BafA1) and chloroquine (CQ)) degradation (Fig. 3g), indicating that the proteasome is important for NBEAL1 degradation. LC3 was used as a positive control for inhibition of autophagy through BafA1 and CQ-treatment. During autophagy, a cytosolic version of LC3 (LC3-I) is conjugated to phosphatidylethanolamine in the autophagosomal membranes (LC3-II) and accumulates upon inhibition of lysosomal degradation (Fig. 3g).

**Figure 4:**
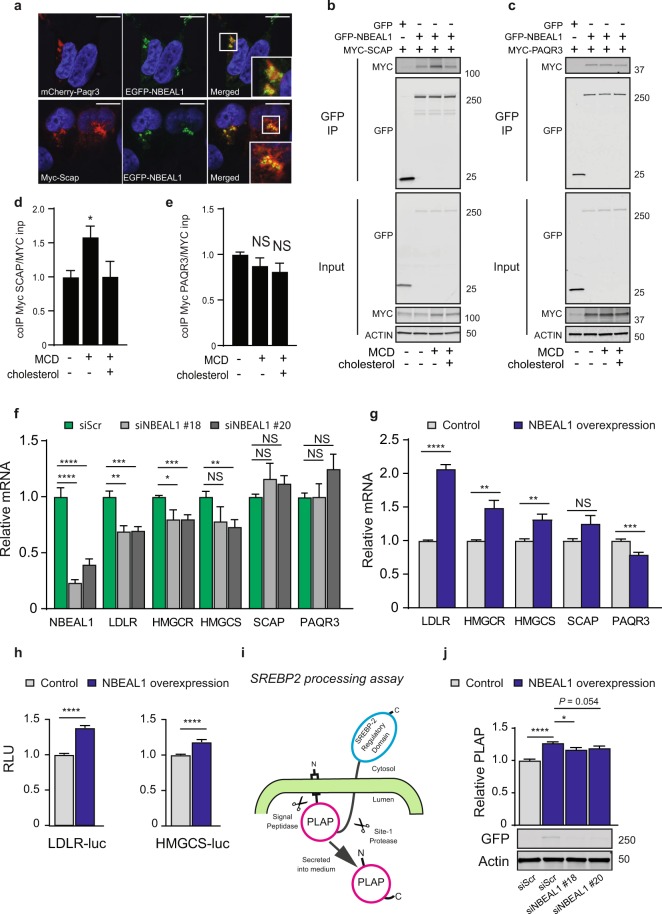
NBEAL1 modulates SREBP2 activity and processing. (**a**) EGFP-NBEAL1, MYC-SCAP and/or Cherry-PAQR3 were transiently transfected into U2OS cells followed by immunofluorescence staining with MYC antibody. Scale bars, 10 µm (N = 3). (**b**) HEK-293T cells expressing EGFP or EGFP-NBEAL1 were transiently transfected with MYC-SCAP. Cells were grown in complete medium (control) or sterol-depleted for 2 h using 0.5% MCD followed by 2 h restimulation with cholesterol or not. Cell lysates were immunoprecipitated with anti-GFP beads, followed by western blotting with the indicated antibodies. (N = 3). (**c**) HEK-293T cells expressing EGFP or EGFP-NBEAL1 were transiently transfected with MYC-PAQR3. Cells were grown in complete medium (control) or sterol- depleted for 2 h (MCD), followed by 2 h restimulation with cholesterol or not. Cell lysates were immunoprecipitated with anti-GFP beads. (**d,e**) Interactions between co- immunoprecipitated (**d**) EGFP-NBEAL1 and MYC-SCAP or (**e**) EGFP-NBEAL1 and MYC- PAQR3 were related to MYC input and presented as relative to control (N = 3). Bars represent mean ±S.E.M. *P < 0.05. NS, not significant by Student’s t-test. (**f**) Expression levels measured by qPCR in primary HUVECs depleted of NBEAL1 using two different siRNAs or (**g**) EGFP- NBEAL1 HEK-293T cells treated or not with doxycycline. All cells were starved for cholesterol prior to harvest (N = 3). All bars show mean ± S.E.M. *P < 0.05, **P < 0.01, ***P < 0.001, ****P < 0.0001 by Student’s t-test; NS, not significant. (**h**) HEK-293T expressing EGFP-NBEAL1 cells were transfected with *Ldlr*- or *Hmgcr*-driven luciferase reporters containing SREs. The Renilla luciferase reporter pRL-CMV was used as internal control. Dual luciferase reporter assays were performed 24 hours post transfection. Data are presented as mean ± SEM (n = 3). Differences are shown as ***p < 0.001 by Student’s t-test. (**i**) Schematic overview of the SREBP2 processing assay (PLAP, placental alkaline phosphatase). (**j**) SREBP2 processing assay in EGFP-NBEAL1 expressing HEK-293T cells with or without depletion of NBEAL1 by siRNA. PLAP was measured in media and related to Renilla values (N = 3). Relative levels of PLAP are shown ± S.E.M. *P < 0.05, ****P < 0.0001 by Student’s t-test.

Given the inhibitory effect of NBEAL1 knockdown on LDLR expression and LDL uptake (Fig. 2), we asked whether overexpression of NBEAL1 in HEK-293T cells, which respond well to cholesterol depletion, would have an opposite effect. Indeed, LDLR levels were higher in HEK-293T expressing EGFP-NBEAL1 as compared to control cells upon cholesterol starvation (Fig. 3h,i). Uptake of cholesterol through the LDLR is tightly regulated and integrated with *de novo* synthesis at the transcriptional levels by the sterol regulatory element-binding protein 2 (SREBP2) transcription factor and its interacting proteins; sterol regulatory element-binding protein cleavage-activating protein (SCAP), insulin-induced gene (Insig) and the recently described progestin and adipoQ receptor family memeber 3 (PAQR3)^32,33^. When cellular levels of cholesterol are high, SREBP2 is retained in the ER bound in a complex with SCAP and Insig. When cellular cholesterol levels drop, SCAP-SREBP2 dissociates from Insig and trafficks to the Golgi, where the two proteases site-1 protease (S1P) and site-2 protease (S2P) promote SREBP2 processing, leading to translocation of the mature form of SREBP2 to the nucleus where it induces the expression of genes involved in cholesterol uptake and synthesis, including LDLR, hydroxy-3-methylglutaryl-CoA reductase (HMGCR) and HMGC synthase (HMGCS). Accordingly, more cholesterol is produced and feeds back to keep the Insig:SCAP:SREBP2 complex in the ER. PAQR3 is a Golgi-anchored transmembrane protein that was found to retain SCAP:SREBP2 in the Golgi and promote SREBP2 maturation^32^. As NBEAL1 localizes to the Golgi (Fig. 3b) where SREBP2 processing occurs, we tested if NBEAL1 could interact with the SREBP2 regulatory proteins, PAQR3 and SCAP. EGFP-NBEAL1 partly co-localized with MYC-SCAP and Cherry-PAQR3 in the Golgi (Fig. 4a) and interacted with both MYC-SCAP and MYC-PAQR3 in co-immnoprecipitation assays (Fig. 4b–e). Most interestingly, the interaction between EGFP-NBEAL1 and MYC-SCAP increased in HEK-293T cells subjected to cholesterol starvation and was reversed upon cholesterol replenishment, with no effect on their expression levels, implying that this interaction is regulated by cholesterol (Fig. 4b,d). In contrast, neither the Golgi localization nor the interaction between EGFP-NBEAL1 and MYC-PAQR3 were affected by cholesterol levels (Fig. 4c,e), suggesting that these proteins form a stable complex. Unlike PAQR3, NBEAL1 does not contain any transmembrane domain. The domain(s) of NBEAL1 needed for its Golgi-association or the interaction with PAQR3 is not known. PAQR3 was recently reported to modulate ER-to-Golgi transport of COPII vesicles by interacting with the WD domains of Sec13 and Sec13A^34^. To test if the WD domain of NBEAL1 interacted with PAQR3, we performed co-IP experiments with MYC-PAQR3 and the following EGFP-NBEAL1 domain modular constructs; PH-BEACH-WD40, BEACH-WD40 or only the BEACH domain. Full length EGFP-NBEAL1 was used as a positive control and interacted with MYC-PAQR3 as expected. Interestingly, all NBEAL1 domain modular proteins interacted with MYC-PAQR3, indicating that the interaction between PAQR3 and NBEAL1 is dependent on the BEACH domain of NBEAL1 rather than the WD40 domain (Supplemental Fig. 6). This suggests that NBEAL1 does not compete with Sec13 and Sec13A for the interaction with PAQR3.

Because NBEAL1 interacted with SREBP2 regulatory proteins, we next asked if NBEAL1 could modulate the transcriptional activity of SREBP2. Indeed, depletion of NBEAL1 by two different siRNA oligos in cholesterol starved primary endothelial HUVECs reduced the gene expression levels of LDLR, HMGCR and HMGCS, but had no effect on expression of genes involved in SREBP2 maturation, including SCAP and PAQR3 (Fig. 4f). Conversely, elevated expression of SREBP2 target genes was seen in cholesterol-starved HEK-293T EGFP-NBEAL1 expressing cells (Fig. 4g). We further examined the effect of NBEAL1 on SREBP activation using luciferase reporter constructs driven by *Ldlr*- and *Hmgcr*-promoters. These promoters contained binding sites for SREBP, so-called sterol regulatory binding elements (SREs), and are mainly regulated by SREBP2. Interestingly, overexpression of NBEAL1 elevated the SRE activity on these promoters, supporting that NBEAL1 induces SREBP2 activity (Fig. 4h). Further supporting a role for NBEAL1 in regulation of SREBP activation, we found that NBEAL1 also had an effect on activation of SREBP1 (Supplemental Fig. 7a–c), which induce the transcription of genes involved in fatty acid synthesis and is regulated by SCAP and PAQR^32^.

To examine whether NBEAL1 could interfere with SREBP2 processing we used a PLAP-BP2 assay^35^. CMV-PLAP-BP2 encodes placental alkaline phosphatase (PLAP) and the regulatory domain of SREBP2 (amino acid 513–1141, BP2) that is processed in a way mimicking endogenous SREBP2 processing, resulting in release of PLAP to the media, which can be measured by luminescence (Fig. 4i). Interestingly, the PLAP level (indicating SREBP2 processing) was significantly higher in HEK-293T EGFP-NBEAL1 cells than in control cells and could be reduced by co-depletion of NBEAL1 (Fig. 4j, Supplemental Fig. 7d), indicating that NBEAL1 affects SREBP2 activity by modulating SREBP2 processing.

## Conclusion

NBEAL1 is a poorly understood protein with no reports on its cellular function or physiological relevance. The characterization of NBEAL1 knockout mice will be important to understand the physiological role of NBEAL1. Herein, we identify human genetic variants in NBEAL1 associated with reduced NBEAL1 expression in arteries and increased risk of CAD. We show that NBEAL1 is a Golgi-associated protein that affects cellular cholesterol metabolism and LDL uptake by modulating SREBP2 activity and processing in primary endothelial cells and HEK-293T expressing NBEAL1 cells. Low expression of NBEAL1 may therefore affect LDL uptake and accordingly LDL levels, which is a risk factor of CAD. Deregulated cholesterol homeostasis promotes the pathology of atherosclerosis, myocardial infarction and strokes. Thus, characterization of NBEAL1 as a novel player involved in regulation of cholesterol homeostasis may provide early diagnosis of persons at risk or pave the way for therapeutic treatment.

## Materials and Methods

### Materials

The following antibodies were used: GFP (Clontech #632381 western blot (WB), 1:3,000), MYC (WB, Abcam # ab9132, 1:5,000), FLAG (WB, Sigma # F1804, 1:500), Lamin A (WB, Sigma-Aldrich # L1293 1:1,000), α-Tubulin (WB, Sigma-Aldrich # T5168, 1:20,000), β-Actin (WB, Cell Signaling Technology # 3700, 1:10,000), GAPDH (WB, Cell Signaling Technology # 5174, 1:5000), NFκB p65 (WB, Santa Cruz Biotechnology # sc-372, 1:1000), VCAM-1 (WB, R&D # BBA19, 1:1000), E-selectin (WB, Santa Cruz Biotechnology # sc-14011, 1:1000), LDLR (WB, BioVision/AH # 3839-100, 1:2,000), Giantin (immunofluorescence (IF), Covance # PRB-114C, 1:500). Fluorophore-conjugated secondary antibodies were from Thermo Fisher Scientific (IRDye® 800CW Donkey anti-Rabbit # SA5-10044, IRDye® 800CW Donkey anti-Mouse SA5- 10170) and LI-COR (IRDye® 800CW Donkey anti-Goat # 926-32214, IRDye® 680RD Goat anti-Rabbit # 926-68071 D). All secondary antibodies were diluted 1:10,000. The following experimental materials were used: FBS (Sigma-Aldrich), penicillin-streptomycin (Termo Fisher Scientific), doxycycline (Clonetech), blasticidin (Termo Fisher Scientific), brefeldin A (Sigma- Aldrich) hygromycin B gold (Sigma-Aldrich), Methyl-beta-cyclodextrin (Sigma-Aldrich), cholesterol (Sigma-Aldrich), IL-1β (R&D # 201-LB- 025), Dual Luciferase® reporter assay system (E1960) was purchased from Promega (Madison, WI, USA).

### Plasmids and transfection for ectopic expression

NBEAL1 was cloned using cDNA library made by reverse transcription (Biorad iScript) from mRNA isolated from U2OS cells. Full-length NBEAL1 was amplified in four regions that were assembled and inserted into pENTR1A (Gateway, Invitrogen) using the Geneart Seamless Plus Cloning and Assembly kit (Thermo Fisher Scientific, # A14603). The cloned full-length sequence was identical to NM_001114132.1 (9058 base pairs), but lacked base pair 639–848, corresponding to exon 5–6 (amino acids 10–172) in the predicted sequence. NBEAL1 variants with alternatively spliced N-terminal region were identified by PCR of the region between nucleotides 48–1296 or 52–1489 (of NM 001114132.1) from cDNA of U2OS cells, which were cloned with Zero Blunt TOPO PCR Cloning Kit (ThermoFisher Scientific), followed by sequencing of the obtained constructs. Tagged NBEAL1 constructs were generated by Gateway LR cloning (Invitrogen) into respective pDEST vectors (Invitrogen). The pRL-CMV vector was from Promega and the pCMV-PLAP-BP2 was a kind gift from Prof. Andrew Brown^34^. MYC-tagged human PAQR3 and MYC-tagged human SCAP was kindly provided from Prof. Yan Chen^30^. Luciferase reporter plasmids containing SREs were kindly provided by Prof. Hitoshi Shimano^36^. The truncated NBEAL1 constructs; EGFP-PH-BEACH-WD40 (aa 1815–2694), EGFP-BEACH-WD40 (1969–2694), and EGFP-BEACH (aa 1991–2284) were generated by PCR amplification from full length EGFP-NBEAL1. The mCherry-PAQR3 plasmid was created by PCR amplification from the cDNA of interest using primers containing KpnI and NotI restriction sites. All plasmids were sequenced to ensure correct reading frame (GATC Biotech). Details on the construction of plasmids are available upon request. Cells were transfected with plasmids encoding EGFP-NBEAL1 variants using FuGene (Roche) or Lipofectamine 2000 (Invitrogen) before further treatment as described.

### Cell culture

Human embryonic kidney (HEK)-293T and U2OS cells were from American Type Culture Collection and were maintained in Dulbecco’s modified Eagle’s medium (Gibco) supplemented with 10% fetal bovine serum (FBS), 5 U ml^−1^ penicillin and 50 μg ml^−1^ streptomycin. FlpIn T- Rex™ HEK-293T and FlpIn T-Rex™ U2OS cells with stable inducible expression of EGFP- NBEAL1 were induced with 10–500 ng/mL doxycycline for 24 h before harvest.

Primary human endothelial cells (HUVEC) were isolated as described from infant umbilical vein of delivering mothers at Rikshospitalet University Hospital^35^. None of the mothers providing umbilical cords had any pregnancy complications. Written informed consent was obtained from each donor and ethical approval for the use of HUVEC was obtained from the Norwegian National Research Ethics Committee for medical and health research (REK 2013/2123). All methods were performed in accordance with the relevant guidelines and regulations. The cells were established at 37 °C and 5.0% CO_2_ in MCDB 131 medium (Sigma) containing 5 mM glucose and supplemented with 7% heat inactivated fetal calf serum (FCS, Sigma), basic fibroblast growth factor (1 ng/mL, R&D), epidermal growth factor (10 ng/mL, R&D), hydrocortisone (1 µg/mL, Sigma), gentamicin (50 µg/mL, GIBCO Invitrogen) and fungizone (250 ng/mL, GIBCO Invitrogen). Cells were used for experiments within three passages, and culture medium was changed every 48–72 h. The purity of the endothelial cell cultures was verified by microscopic observations of each culture as well as regular staining for the endothelial cell marker von Willebrand factor (vWF). For siRNA experiments, the cells were seeded at 1.5 × 10^5^ cells per well in a six well dish for three days knockdown with reverse transfection. A pool of minimum four individual donors was used in each replicate. Mycoplasma testing was done in the core facility of The Norwegian Center for Stem Cell Research and all cell lines were negative for mycoplasma.

### RNA isolation and qPCR

RNA was isolated from cells using the RNeasy plus kit (Qiagen). For cDNA synthesis, 500 ng of extracted RNA was reverse transcribed using SuperScript III (Invitrogen) and random hexamer primers. qPCR was performed with 1 μl of the cDNA synthesis reaction using Kapa SYBR FAST qPCR Master Mix (KapaBiosystems). Primers used to amplify genes of interest were; NBEAL1 (5′-TGCGACTGCCTATCCATTGT-3′, 5′-GACCTGAACGCATCTCAGCA-3′), LDLR (5′-AGTGTGACCGGGAATATGACT-3′, 5′-CCGCTGTGACACTTGAACTT-3′), HMGCR (5′-GTTAACTGGAGCCAGGCTGA-3′, 5′-CCTTGGATCCTCCAGATCTCAC-3′), HMGCS (5′-TGTCCTTTCGTGGCTCACTC-3′, 5′-GGTGAAAGAGCTGTGTGAAGG-3′), PAQR3 (5′-AGCGGTACTTTCCAGGACAAC-3′, 5′-ACTGTTGACTGATGCCACCAA-3′), SCAP (5′-ACCTGTGGAATTCACCACCC-3′, 5′-ACCCACATACCACTCAGGCT-3′), FAS (5′-CTTCAAGGAGCAAGGCGTGA-3′, 5′-ACTGGTACAACGAGCGGATG-3′), ACACA (5′-GCCTCTCAGAGACAACGTGA-3′, 5′-GAGAATCTGACCAGCTGAGAGG-3′), ACL (5′-GACTTCGGCAGAGGTAGAGC-3′, 5′-AGGAGTTCTTTGCCCGTCTG-3′), ELOVL6 (5′-CAAAGCACCCGAACTAGGAGA-3′, 5′-GGAGCACAGTGATGTGGTGA-3′), SCD1 (5′-ACACCCAGCTGTCAAAGAGA-3′, 5′-GCCAGGTTTGTAGTACCTCCTC-3′), TBP (5′-TTGTACCGCAGCTGCAAAAT-3′,5′-TATATTCGGCGTTTCGGGCA-3′). All target transcripts were normalized to Tata-binding protein (TBP) and analysed using the comparative *C*_T_(ΔΔ*C*_T_) method. RNA data are presented as mean and S.E.M. for three independent experiments.

### siRNA experiments

The following siRNA oligonucleotides were used: Dharmacon ON-TARGET Plus; NBEAL1- J-031856-18 GGAUAUAACAGCUAGAGUA, NBEAL1-J-031856-20 UCAAACAUGUGGACCGAGA and On-Target plus Control siRNA D-001810-01-50; and Thermo Fisher Silencer® Select RELA (# s11914). 5–25 nM of siRNA was delivered to the cells by reverse transfection using Lipofectamine 2000 RNAi max (Invitrogen). Cells were harvested 72 h post transfection for downstream assays and knockdown efficiency was validated by qPCR or immunoblotting.

### Cell lysis and immunoprecipitation

Whole-cell extracts were prepared in precipitation assay buffer (50 mM Tris/HCl pH 7.4, 150 mM sodium chloride, 1% NP-40, 0.5% sodium deoxycholate, 0.1% SDS, 2 mM EDTA) supplemented with protease inhibitor cocktail (Roche). Nuclear and post-nuclear supernatant fractions were prepared using the NE-PER extraction kit (Pierce Biotechnology). Protein concentration was measured by Biorad Protein Assay to run equal amounts of cell lysate on SDS–polyacrylamide gel electrophoresis (PAGE), followed by western blotting using indicated primary antibodies and fluorophore-conjugated secondary antibodies. Detection and analysis was performed by LI-COR Odyssey imaging. For immunoprecipitation from lysates, EGFP, EGFP-NBEAL1, MYC, MYC PAQR3 were immunoprecipitated by GFP (Chromotek) following the manufacturer’s protocol. The resulting immunoprecipitates or pulldowns were separated by SDS–PAGE and analysed by western blotting.

### Proteinase K protection assay

HEK-293T EGFP-NBEAL1 cells treated with doxycycline were seeded in 10 cm dishes and harvested in a HES buffer (15 mM Hepes KOH pH 7.4, 1 mM EDTA, 250 mM Sucrose) without protease inhibitor cocktail. Cells were lysed using a cell cracker (Isobiotec), and centrifuged 1000 × *g* 10 min, 4 °C to remove intact cells, mitochondria and nucleus. The supernatant was transferred to ULTRA centrifuge tubes and centrifuged at 100.000 ×*g* 30 min, 4 °C. The supernatant (cytosolic fraction) was saved membrane samples were re-suspended in HES buffer, HES buffer with 25 µg/mL proteinase K (Roche), or HES buffer with 25 µg/mL proteinase K and 1% Triton X-100. Samples were incubated at room temperature for 20 min. Proteinase K was then inactivated by adding protease inhibitor cocktail and sample buffer.

### Protein degradation assay

EGFP-NBEAL1 expressing HEK-293T cells were treated with or without 10 ng/mL doxycycline for 24 h. Cells were washed three times in warm media to remove doxycycline followed by a 24 h incubation in the presence or absence of 100 nM MG-132, 100 nM Bafilomycin A1 or 50 µM chloroquine.

### Cholesterol starvation/stimulation in cells

The cellular cholesterol levels are tightly regulated at the transcriptional level by SREBP2 and downstream target genes. Cholesterol starvation experiments can be used to induce the cholesterol synthesis. Treatment of cells with MCD is the most common method to deplete cells from cholesterol. MCD has a central cavity that can form a 2:1 complex with free cholesterol 2:1^37^. Thus, the cells are depleted of cholesterol and switches on SREBP2 dependent gene programs for cholesterol synthesis (i.e. HMGCR and HMGCS) and uptake (i.e. LDLR). When the sterol depleted cells are replenished with medium containing excess cholesterol, the cells attempt to restore cellular cholesterol levels within the normal range and switch off the cholesterol synthesis pathway. HUVEC and HEK-293T were rinsed twice in serum free media and was incubated with empty DEMEM containing 0.5% MCD for 2 h. Cells were then transferred to DEMEM containing 0.1% MCD (starved condition) or to DMEM containing 20 μg/ml cholesterol pre-complexed with 0.1% MCD (resulting in MCD:cholesterol at 1:1 molar ratio, 50 μM) and incubated for 4 h. MCD:cholesterol complexes were prepared by diluting a 20 mg/mL cholesterol stock solution (in EtOH) 1000-fold into a 15-ml falcon tube containing DMEM + 0.1% MCD + 0.5% LDS, resulting in 50 μM final concentration of both cholesterol and MCD.

### Indirect Immunofluorescence

Cells were seeded on fibronectin-coated coverslips (10 µg/mL) and transfected with siRNA as described above or with EGFP-NBEAL1 construct 24 h after plating. Cells were, where indicated, treated or not with Brefeldin A for 10 min (5 µg/mL final). Twentyfour hours after transfection, cells were washed twice in PBS and fixed with 4% paraformaldehyde on ice. The cells were then permeabilized in a solution containing 5% FBS and 0.05% saponin (Sigma - Aldrich) in PBS before incubation with primary antibody for 1 h, washed in PBS and incubated with secondary antibodies for 45 min. Cells were washed in PBS and nuclei were counterstained with Hoechst (1:1000 dilution of 10 μg/mL stock) in PBS for 10 min. The coverslips were mounted with ProLong® Gold Antifade Mountant (Thermo Fisher Scientific). Confocal microscopy was performed using Zeiss LSM 710 ELYRA.

### Luciferase reporter assay

HEK-293T expressing EGFP-NBEAL1 cells were seeded in 24-well plates at 1 × 10^5^ cells per well and incubated for 24 h. Cells were transfected with 200 ng of luciferase reporters containing SREs (pGL2-LDLR-luc, pGL2-HMGCS-luc, pGL2-FAS-luc). Renilla luciferase reporter (pRL-CMV; 50 ng) was included as an internal control for transfection efficiency. All transfections were performed with Lipofectamine 2000. Dual luciferase reporter assay was performed 24 hours post transfection as previously described^38^ After 18 hours of incubation, cells were washed with PBS and lysed in Passive Lysis Buffer (Promega, Madison, WI). Dual-Luciferase® Reporter Assays (Promega, #E1960) were run on a Synergy H1 plate reader (BioTek® Instruments, Winooski, VT) according to manufacturer’s manual. Readings of Firefly Luciferase were normalized to the Renilla Luciferase readings, and data from three independent transfections experiments run in duplicates are presented.

### SREBP2-processing assay

Analysis of SREBP-2 processing was measured by a secreted alkaline phosphatase assay as described previously^35^. In brief, HEK-293T expressing EGFP-NBEAL1 cells were seeded in 24-well plates at 1.2 × 10^5^ cells per well and incubated for 24 h. Cells were co-transfected with 0.05 µg pRL-CMV and 0.45 µg pCMV-PLAP-BP2 using Lipofectamine 2000. The pCMV- PLAP-BP2 contains a fusion protein, which codes for placental alkaline phosphatase (PLAP, amino acid 1–506) and the regulatory domain of SREBP2 (BP2, amino acid 513–1141). The PLAP is flanked by a cleavage sites for a signal peptidase and Site-1 protease (S1P) and cleaved by endogenous S1P upon SREBP2 processing. When SREBP2 is processed, the cleavage of both proteases leads to PLAP secretion into the media. PLAP was measured by Phospha-Light™ SEAP Reporter Gene Assay System according to manufacturer’s recommendation (Thermo Fisher Scientific). The cells were immediately lysed in passive lysis buffer (Promega). The transfection level of pRL-CMV was detected using the Stop-Glow component from Dual-Luciferase® Reporter Assay System (Promega) according to manufacturer’s protocol and used as a control for transfected cells.

### LDL uptake

LDL uptake was measured using using fluorescently labelled Low Density Lipoprotein from Human Plasma, BODIPY® FL complex (Thermo Fisher Scientific # L3483) according to manufacturer’s protocol. In brief, HUVECs were reverse transfected with indicated siRNA oligos and seeded at 1 × 10^4^ on gelatin coated chamber slides. Cells were changed to lipid-deprived media containing 2% lipid depleted serum 24 h before harvest. Cells were labelled with 15 µg/mL BODIPY® FL for 30 min on ice and chased for 10 min at 37 °C. Cells were fixed in 4% paraformaldehyde on ice, washed three times in PBS and counterstained with Hoechst in PBS. The intensity was quantified from an average of 500 cells per experiment and each experiment represent cells pooled from four individual donors. For quantitative analysis, cells were imaged on a Zeiss Cell Observer microscope. The Zen Blue software (Zeiss) was used for automated capture of 40 images per sample with a 20x magnification. A pipeline was created in Cellprofiler^39,40^ to count dots and to measure intensity per cell. KNIME was used for data mining and analysis^41^.

### Statistical analysis

All data are presented as means and S.E.M. Two-way analysis of variance (ANOVA) followed by Tukey’s multiple comparison tests, one-way ANOVA followed by Dunnett’s multiple comparison test or two-tailed Student’s t tests were used to assess statistical significance (*P* < 0.05). Statistical tests applied are described in the figure legends. The tissue expression data used for the analyses described in this manuscript were obtained from the GTEx Portal on 11/09/2018. The UCSC Xena browser was utilized for analyzing expression data (http://xena.ucsc.edu/), Kallisto was used for analyzing isoform expression^42^. GWA data from the CARDIoGRAMplusC4D consortium was retrieved from T2D-GENES Consortium, GoT2D Consortium, DIAGRAM Consortium (Type2Diabetes Knowledge Portal) on 3/20/2017, and minor allele frequency reported for Europeans based on data from the 1000 Genomes Project. Linkage disequilibrium (LD) analysis was performed using SNAP^43^. Variants were visualized using the TOPPAR software^44^.

## Supplementary information

Supplementary information.

Supplemental Table 1.

Supplemental Table 2.

Supplemental Table 3.

Supplemental Table 4.

Supplemental Table 5.
