# Supplementary material for: NBEAL1 controls SREBP2 processing and cholesterol metabolism and is a susceptibility locus for coronary artery disease
Source: Sci Rep. 2020 Mar 11;10:4528. doi: 10.1038/s41598-020-61352-0 (PMC7066131; doi:10.1038/s41598-020-61352-0)
Supplement: Supplementary file 1 — Supplementary information. [file 41598_2020_61352_MOESM1_ESM.pdf]

Supplementary information

NBEAL1 controls SREBP2 processing and cholesterol metabolism and is a susceptibility locus for coronary artery disease

Christian Bindesboll[1*], Aleksander Aas[1], Margret Helga Ogmundsdottir[2], Serhiy Pankiv[1], Trine Reine[3], Roberto Zoncu[4], Anne Simonsen[1*#]

[1] Department of Molecular Medicine, Institute of Basic Medical Sciences and Centre for Cancer Cell Reprogramming, Institute of Clinical Medicine, Faculty of Medicine, University of Oslo, 1112 Blindern, 0317 Oslo, Norway, [2] Department of Biochemistry and Molecular Biology, Biomedical Center, Faculty of Medicine, University of Iceland, Vatnsmyrarvegur 16, 101 Reykjavik, Iceland, [3]Department of Nutrition, Institute of Basic Medical Sciences, University of Oslo, 1112 Blindern, 0317 Oslo, Norway, [4]Department of Molecular and Cell Biology, University of California, Berkeley, Berkeley, CA 94720, USA.

Running title: *NBEAL1 is associated with increased risk of coronary artery disease*

Supplemental Figure 1

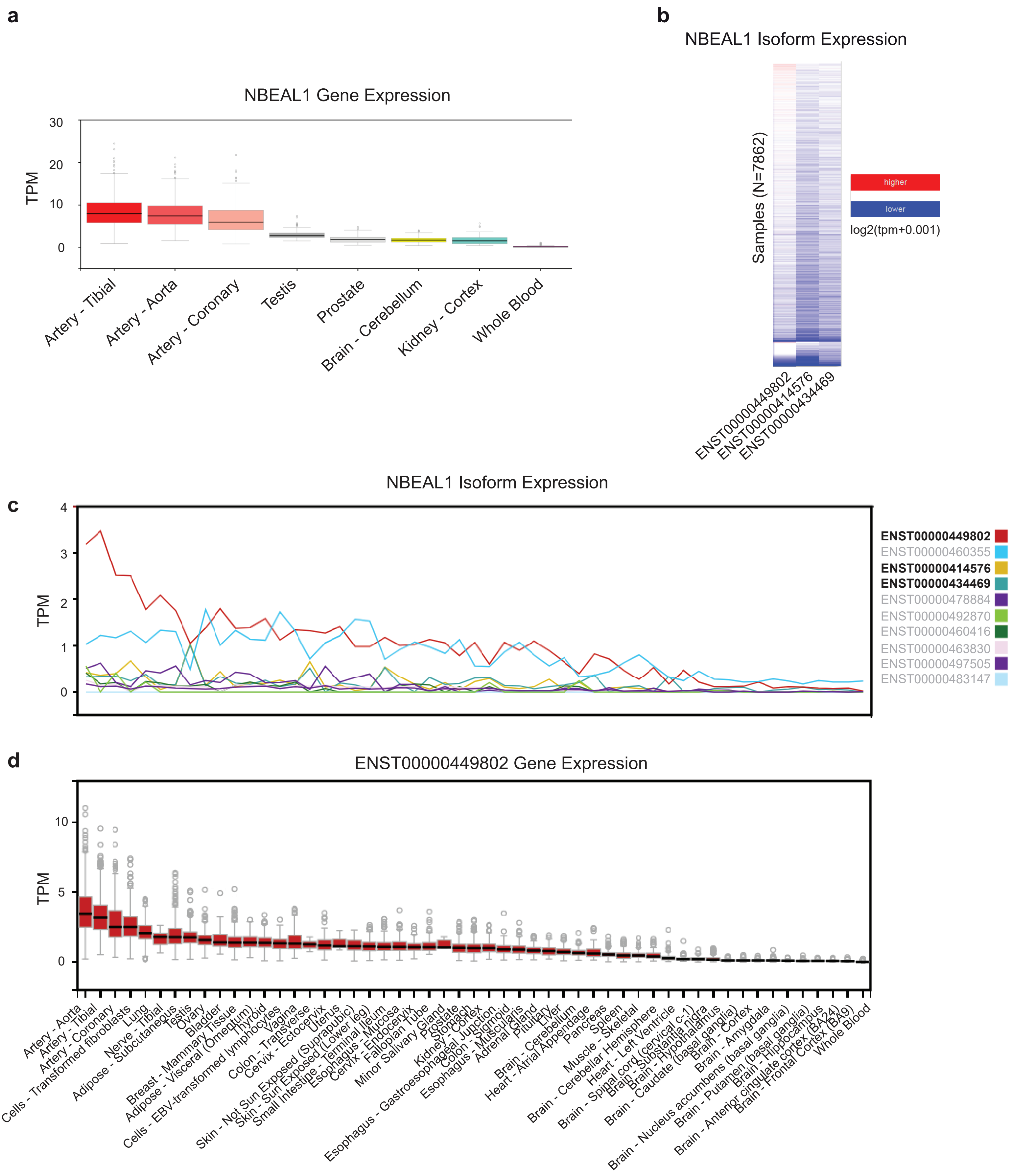

Supplemental Fig. 1. Expression profile of NBEAL1. Related to Figure 1. (a) RNA sequencing data obtained from the GTEx Portal database showing expression values of NBEAL1 displayed as TPM (transcript per million) in different human tissues. (b) Expression of the three protein coding isoforms of NBEAL (ENST00000449802, ENST00000414576, ENST00000434469) in all tissues in the GTEx database, analyzed by the Xena browser utilizing Kallisto. Values are displayed as log2 (tpm+0.001); each row represents one of the 7,862 samples analyzed. (c) Expression levels of different NBEAL1 isoform transcripts in various tissues according to the GTEx database. Protein coding isoforms are shown in bold (ENST00000449802,

Supplemental Figure 2

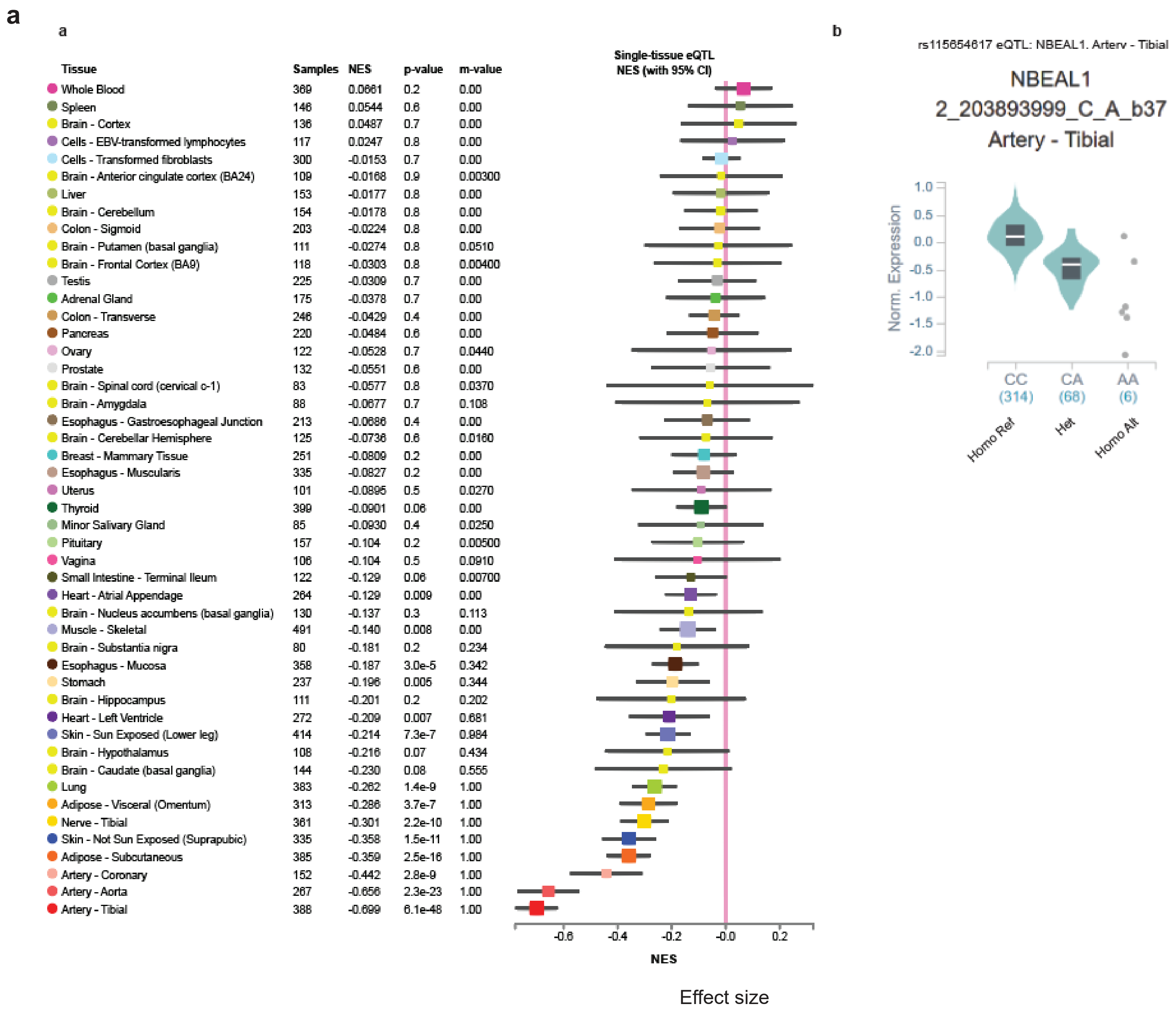

Supplemental Fig. 2. NBEAL1 variant expression in association with CAD. Related to Figure 1. (a) The variant of NBEAL1 rs115654617 is associated with decreased gene expression of NBEAL1 in multiple human tissues. Data obtained from GTEx. (b) eQTL (expression quantitative trait loci) of the NBEAL1 variant rs115654617 with NBEAL1 expression levels in Artery-Tibial, data obtained from GTEx, homozygous reference noncarriers (Homo Ref (N=314)), heterozygous (Het (N=68)) or homozygous alternate (Homo Alt) for the minor allele variant (N=6). (e) NBEAL1 expression in Artery Tibial vs OR of CAD for 281 variants associated with CAD. Correlation was analyzed using Spearman

Supplemental Figure 3

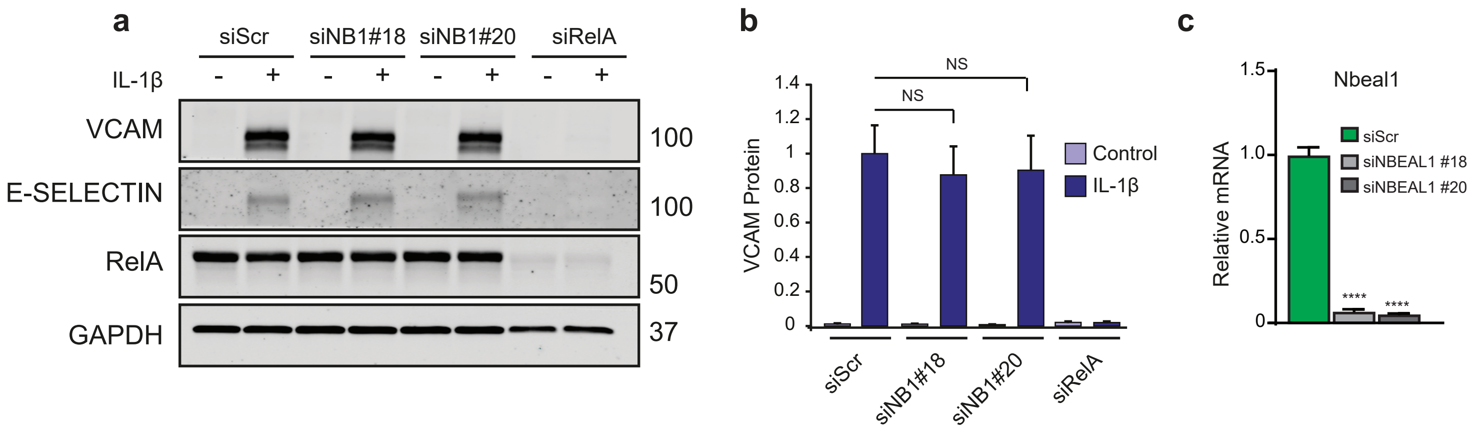

Supplemental Fig. 3. NBEAL1-depletion is does not regulate IL-1β dependent expression of monocyte recruiting proteins VCAM and E-Selectin. (a) HUVEC cells were depleted or not of NBEAL1 and treated with 5 ng/mL IL-1β for 4 h followed by western blotting with indicated antibodies (N=3). siRNA targeting RelA was used as a control. (b) Quantification of VCAM protein levels relative to GAPDH in HUVECs from three independent experiments. All bars show mean ± S.E.M. Statistical differences between IL-1β induced expression was analyzed by two-way ANOVA followed by Tukey's multiple comparison test; NS, not significant. (c) Gene expression analysis of NBEAL1 by qPCR revealing NBEAL1 knockdown efficiency from experiments in (a). Bars represent mean ± S.E.M. and one-way ANOVA followed by Dunnett's multiple com-

Supplemental Figure 4

a

	**Start – end**	**Peptide Sequence**
**Approach 1** Monoclonal Antibody	6-19	RLFELWMLYCTKKD
	496-509	TLDLHSSLHQTCAE
	724-737	PSQIPDPPFSSPIT
	985-998	EQVSLEKNMQLLQQ
	1207-1220	HQIINTDPVINFKD
	1366-1379	SNPSHLSLDLSGID
	1510-1523	KTNPVTAENAFRLV
	1752-1765	QENLRYNNMLKQLS
	1936-1949	SQIREIHLRRYNLR
	2136-2149	SIPATWQALMDNPY
	2420-2433	DNSIQVMSLTKGKI
	2681-2694	ISAGETEYNTQDSK
**Approach2** Polyclonal Antibody	1299-1313	STEDTKKNSDEKTDE
	1395-1409	PSTPSPVESTKSFSV
	1726-1740	KRDREGGESKLKFQE
	2671-2685	FWGSSKRLSQISAGE

b

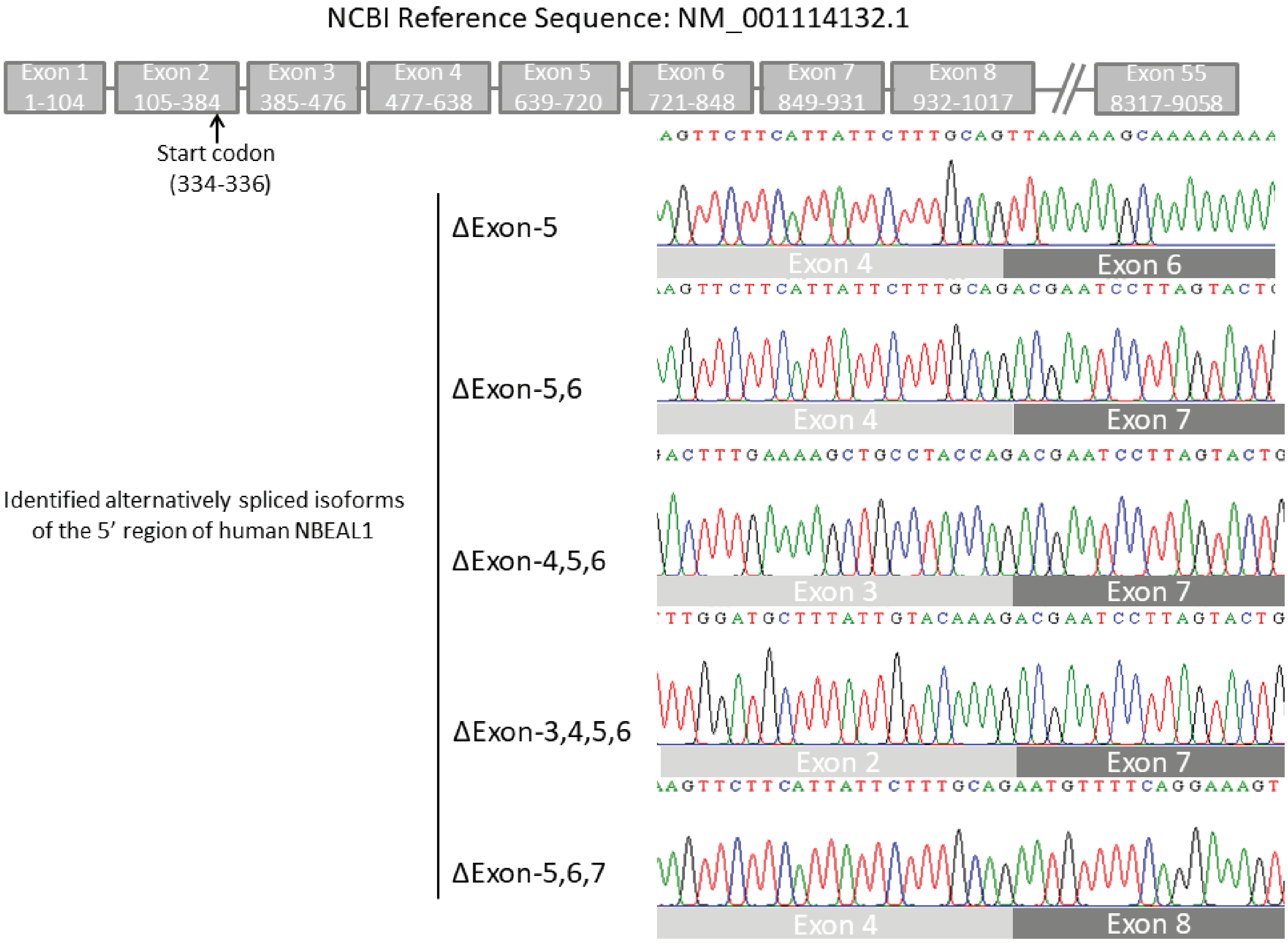

Supplemental Fig. 4. (a) Sequences of NBEAL1 peptides used to generate the antibodies against NBEAL1. (b) Schematic exon structure of the reference mRNA sequence of NBEAL1 (NM 001114132.1) and Sanger sequencing chromatograms of the splicing regions of identified alternatively spliced isoforms of NBEAL1.

Supplemental Figure 5

a

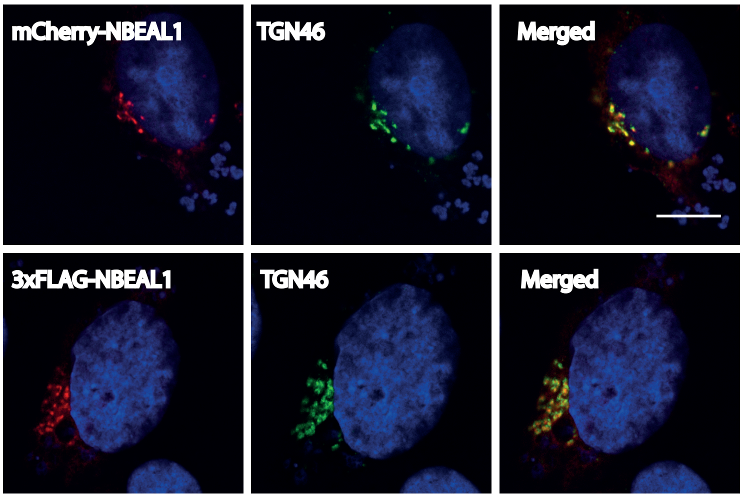

Supplemental Fig. 5. NBEAL1 is a Golgi associated protein. Related to Figure 3. mCherry-NBEAL1 and 3xFLAG-NBEAL1 transiently expressed in U2OS cells were fixed and subjected to immunofluorescence staining of endogenous TGN46 (IF, BIO-RAD #AHP500GT, 1:500). Scale bars, 10 µm (N=3).

Supplemental Figure 6

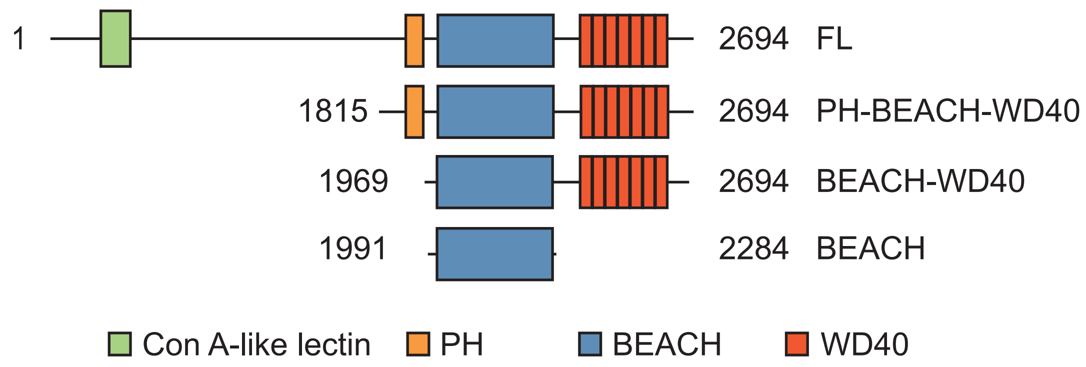

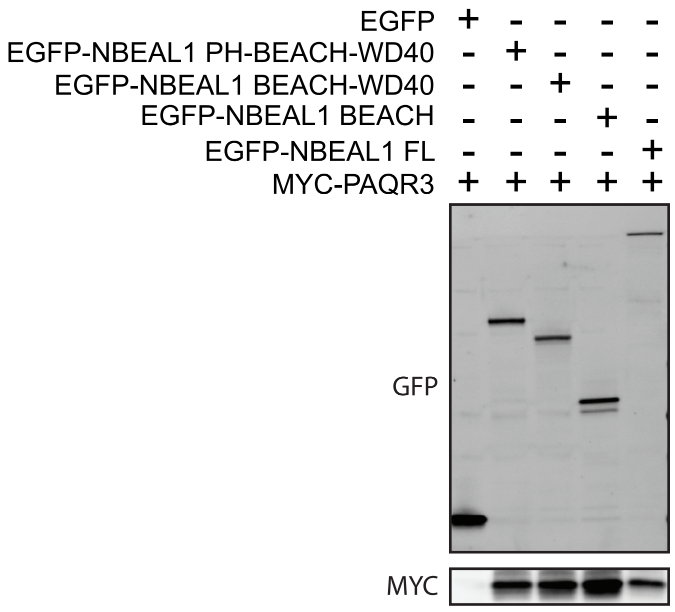

Supplemental Fig. 6. The BEACH domain of NBEAL1 interacts with PAQR3. Schematic representation of the NBEAL1 constructs. Key domains of the proteins are highlighted with boxes including the Concanavalin A (ConA)-like lectin, the pleckstrin homology (PH), BEACH, and WD40 repeat domains. The numbers refer to amino acids. HEK-293T cells were transiently transfected with EGFP, EGFP-NBEAL1 full length (FL) or truncated EGFP-NBEAL1 constructs and MYC-PAQR3. Cell lysates were immunoprecipitated with anti-GFP beads, followed by immunoblotting of the immunoprecipitates with the indicated antibodies. (N=3).

Supplemental Figure 7

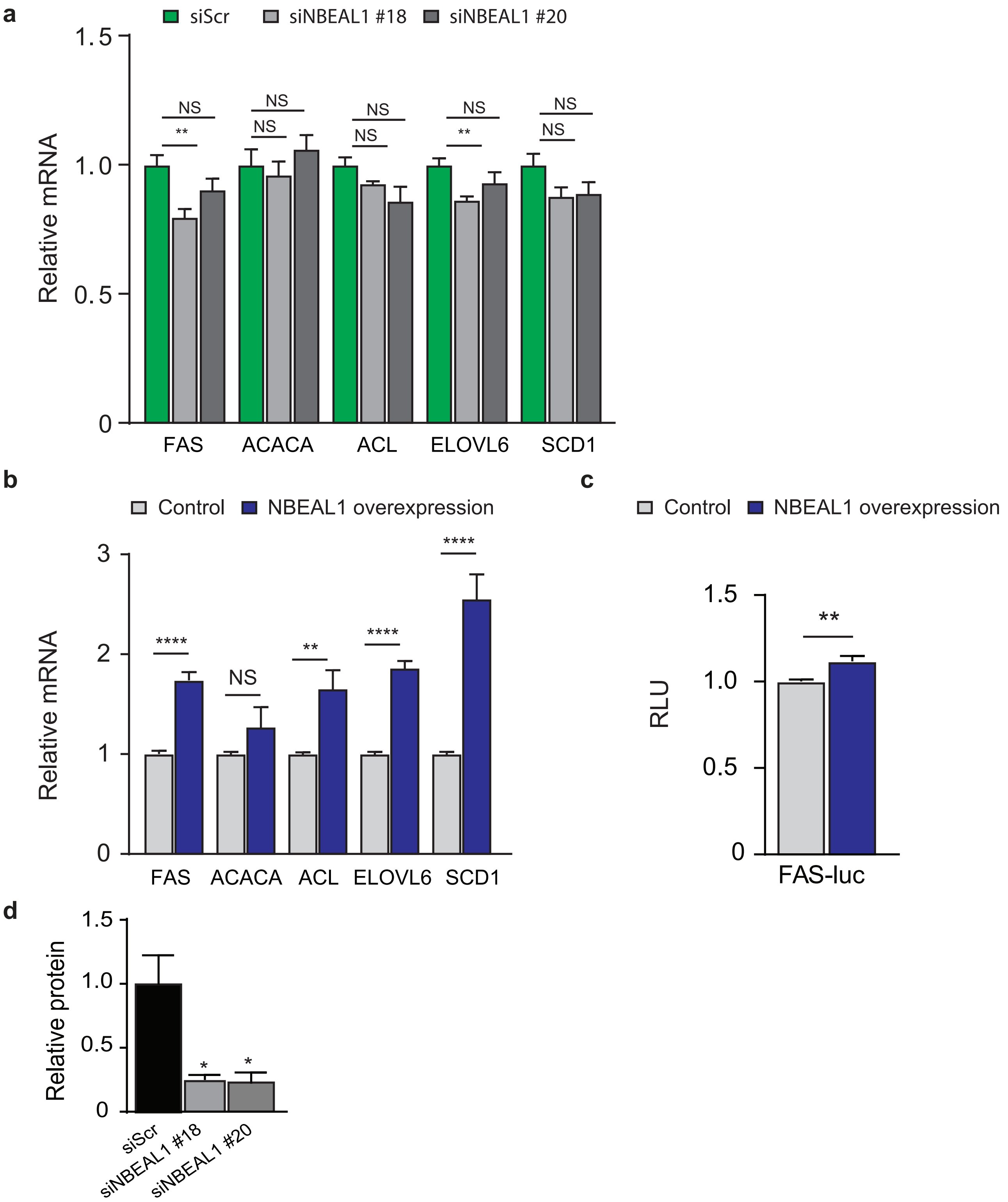

Supplemental Fig. 7. NBEAL1 modulates SREBP1 activity. Related to Figure 4. (a) Expression levels measured by qPCR in primary HUVECs depleted of NBEAL1 using two different siRNAs or (b) EGFP-NBEAL1 HEK-293T cells treated or not with doxycycline. All cells were starved for cholesterol prior to harvest (N=3). All bars show mean ± S.E.M. * $P < 0.05$, ** $P < 0.01$, **** $P < 0.0001$ by Student's t-test; NS, not significant. FAS (FA synthase), ACACA (acetyl CoA carboxylase α), ACLY (ATP citrate lyase), SCD1 (stearoyl CoA desaturase 1), (ELOVL6) (elongation of long-chain fatty acid family member 6). (c) HEK-293T expressing EGFP-NBEAL1 cells were transfected with Fas-driven luciferase reporters containing SREs. The Renilla luciferase reporter pRL-CMV was used as internal control. Dual luciferase reporter assays were performed 24 hours post transfection. Data are presented as mean ± SEM (N=3). Differences are shown as **$P < 0.001$ by Student's t-test. (d) Related to Fig. 4j. Quantification of EGFP-NBEAL1 protein relative to Actin from three independent experiments presented as mean ± S.E.M. Statistical differences were analyzed using one-way ANOVA followed by Dunnett's multiple comparison test relative to siScr. * $P <0.05$.

Unprocessed images from Figure 2

a

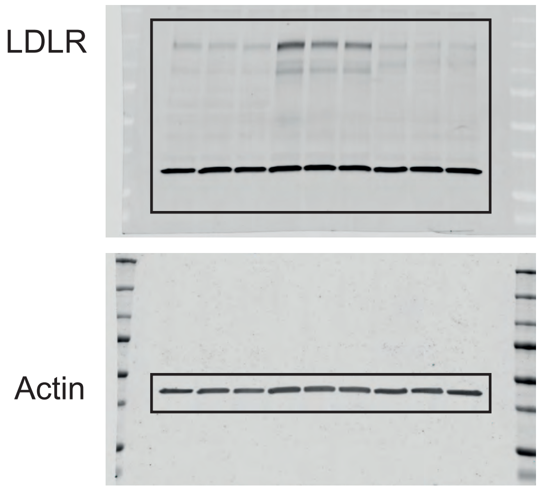

Unprocessed images from Figure 3

c

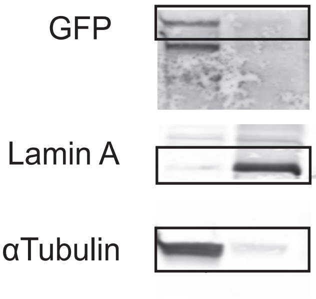

d

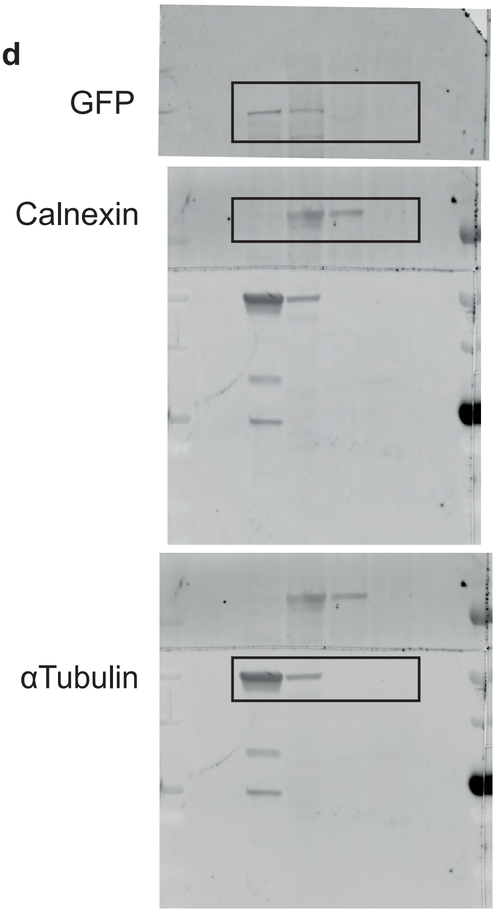

e

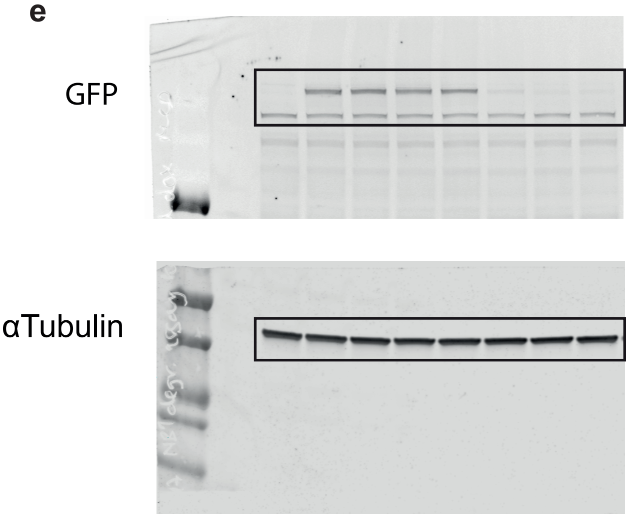

g

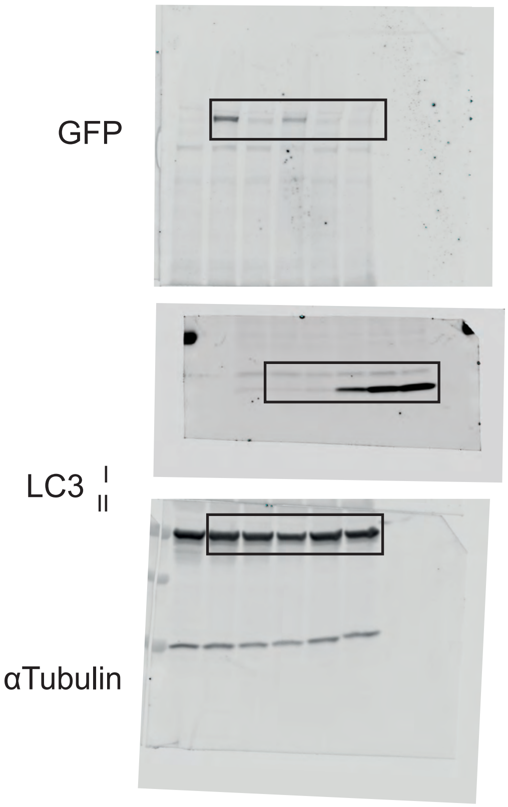

h

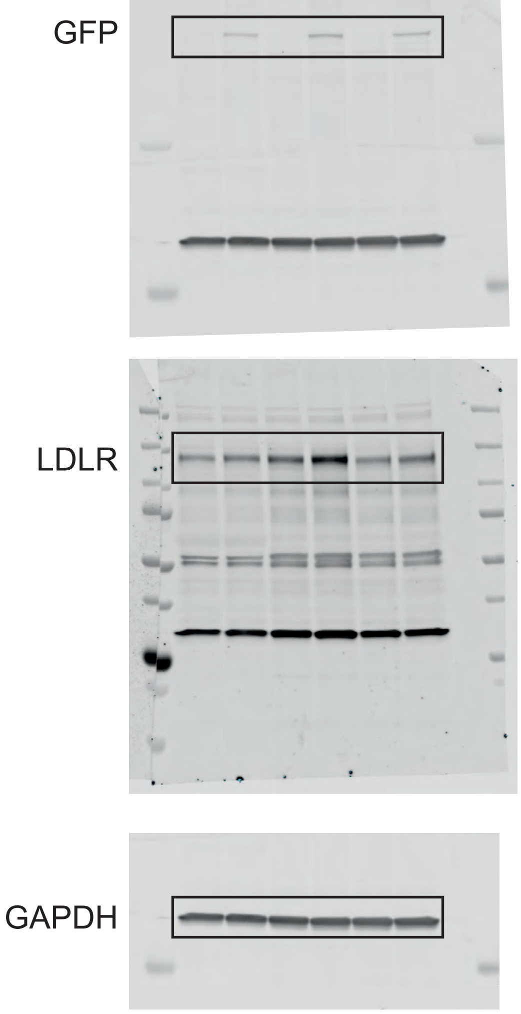

Unprocessed images from Figure 4

b

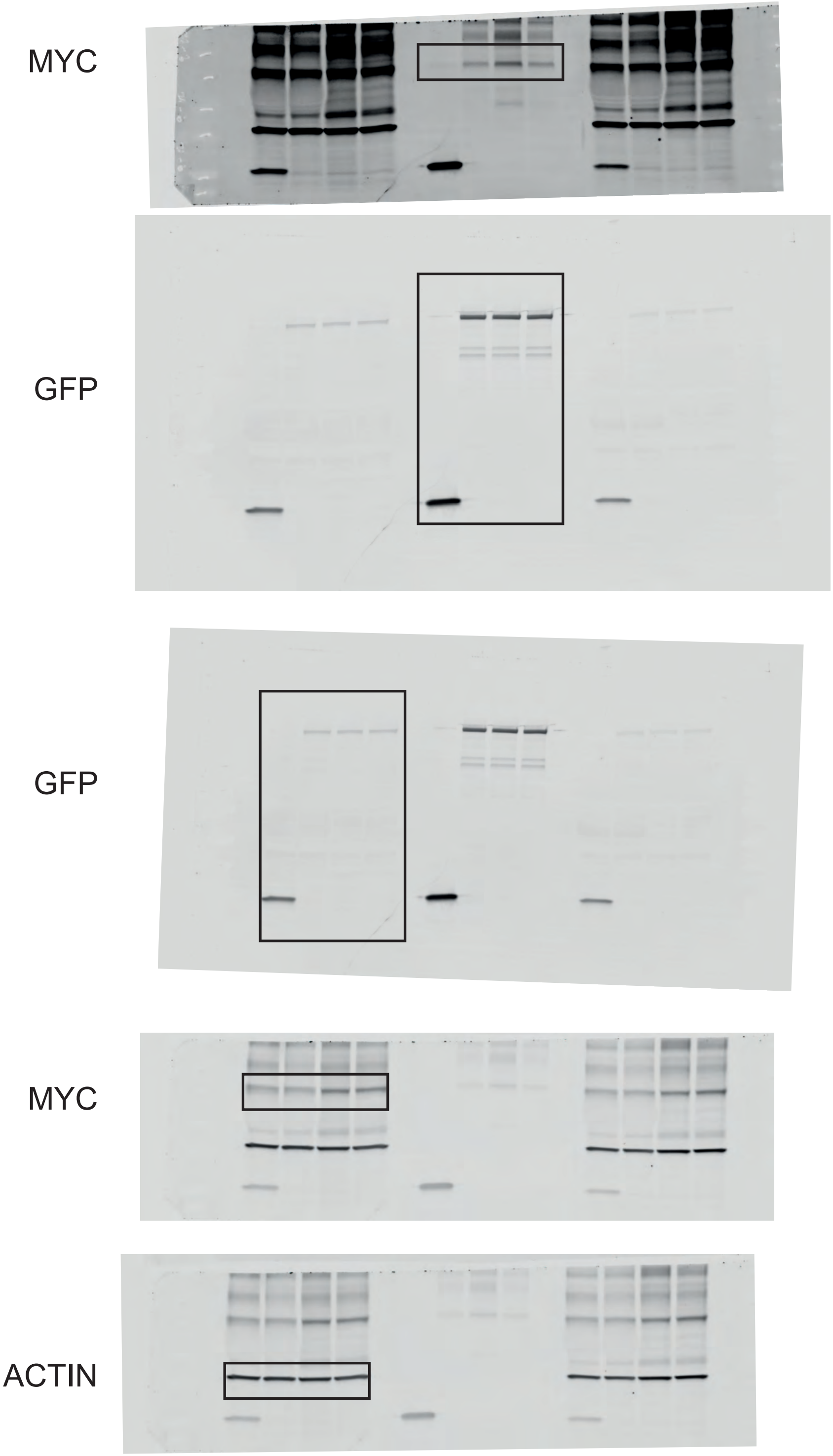

c

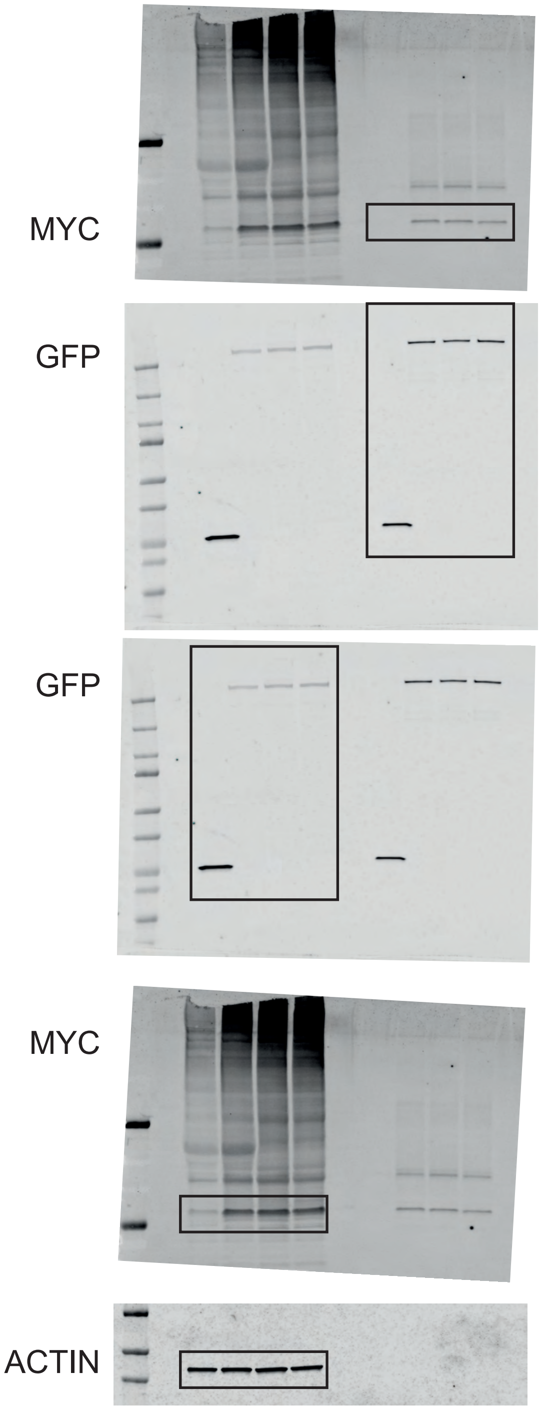

i

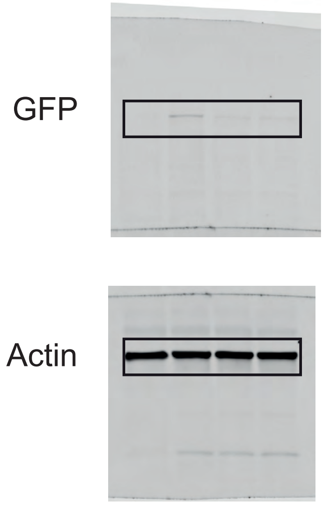

Unprocessed images from Supplemetal Figure 3

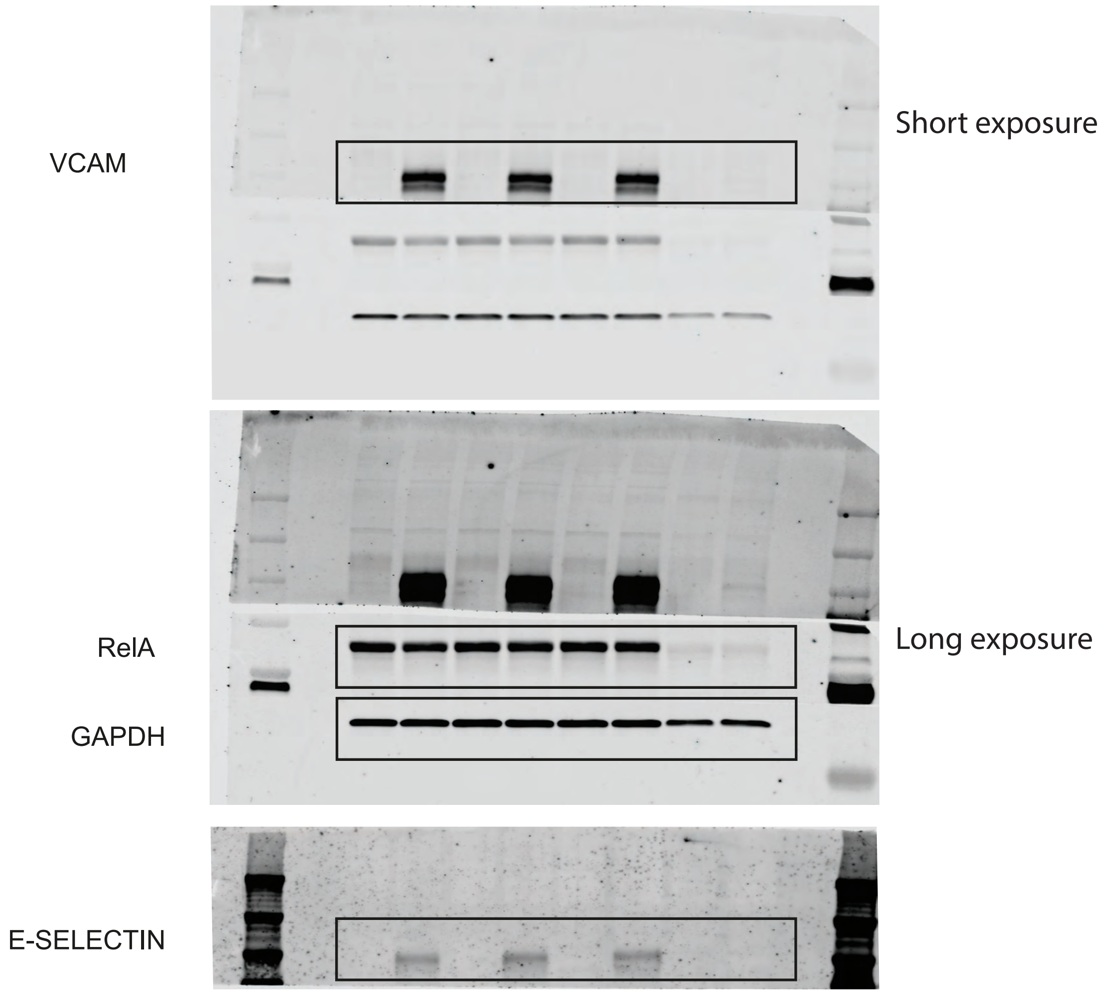

Unprocessed images from Supplemetal Figure 7

GFP

MYC

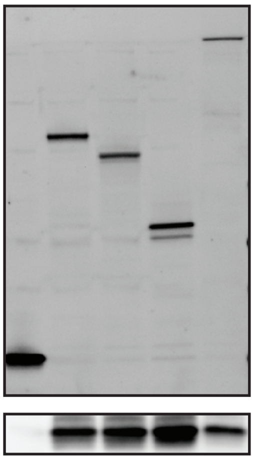

GFP

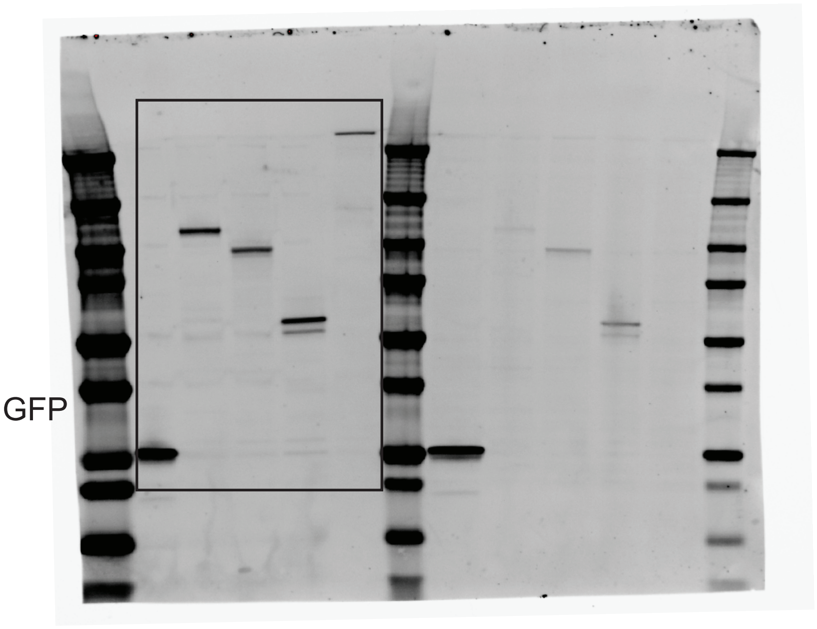

MYC

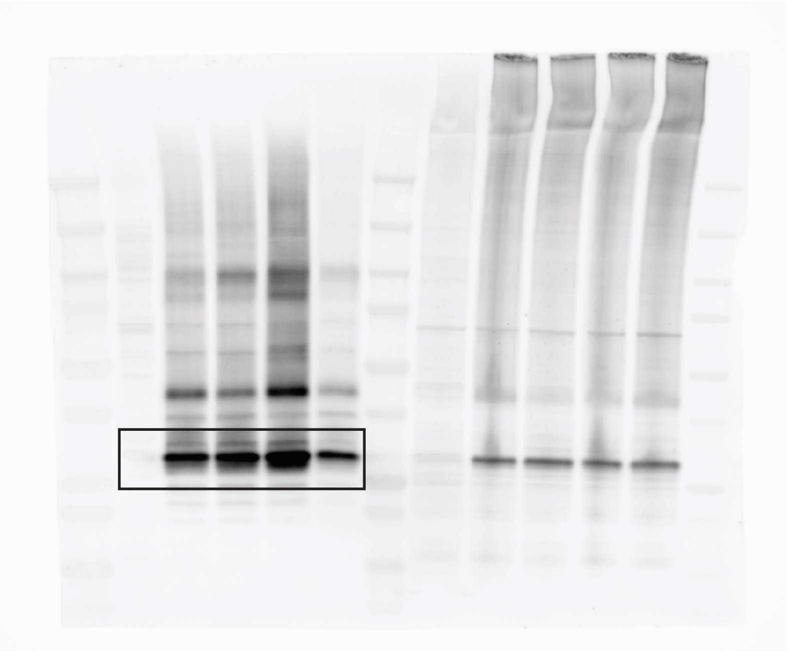

Supplemental Tables

Supplemental Table 1. Complete list of variants on chromosome 2 associated with increased risk of CAD. Related to Figure 1. Separate Excel file.

Supplemental Table 2. Variants in a linkage disequilibrium (LD) with the NBEAL1 variant rs115654617 that associate with CAD. Related to Figure 1. Separate Excel file.

Supplemental Table 3. Comparison of gene expression in rs115654617 carriers and non-carriers. Data obtained from GTEx. Related to Figure 1. Separate Excel file.

Supplemental Table 4. NBEAL1 expression in arteries negatively correlates with CAD-associated chromosome 2 variants. Data obtained from GTEx. Related to Figure 1. Separate Excel file.

Supplemental Table 5. NBEAL1 expression in liver is not affected by CAD-associated chromosome 2 variants. Data obtained from GTEx. Related to Figure 1. Separate Excel file
